# Smoothing sample average approximation method for solving stochastic second-order-cone complementarity problems

**DOI:** 10.1186/s13660-018-1674-2

**Published:** 2018-04-10

**Authors:** Meiju Luo, Yan Zhang

**Affiliations:** School of Mathematics, Liaoning University, Liaoning, China

**Keywords:** 90C33, 65K05, 90C15, Second-order-cone, Stochastic complementarity problems, Sample average approximation, Smoothing function, Convergence

## Abstract

In this paper, we consider stochastic second-order-cone complementarity problems (SSOCCP). We first use the so-called second-order-cone complementarity function to present an expected residual minimization (ERM) model for giving reasonable solutions of SSOCCP. Then, we introduce a smoothing function, by which we obtain a smoothing approximate ERM model. We further show that the global solution sequence and weak stationary point sequence of this smoothing approximate ERM model converge to the global solution and the weak stationary point of the original ERM model as the smoothing parameter tends to zero respectively. Moreover, since the ERM formulation contains an expectation, we employ a sample average approximate method for solving the smoothing ERM model. As the convergence analysis, we first show that the global optimal solution of this smoothing sample average approximate problem converges to the global optimal solution of the ERM problem with probability one. Subsequently, we consider the weak stationary points’ convergence results of this smoothing sample average approximate problem of ERM model. Finally, some numerical examples are given to explain that the proposed methods are feasible.

## Introduction

The second-order-cone (SOC) in $R^{n}$ ($n\geq1$) is defined as follows:

$$ \mathcal{K}^{n}=\bigl\{ (x_{1},x_{2})\in R\times R^{n-1}\mid\|x_{2}\|\leq x_{1}\bigr\} , $$

where $\|\cdot\|$ denotes the Euclidean norm. The second-order-cone complementarity problems (SOCCP) are to find vectors $x, y\in R^{n}$ and $z\in R^{l}$ satisfying

$$ \langle x,y\rangle=0, \qquad x\in\mathcal{K},\qquad y\in\mathcal{K}, \qquad F(x,y,z)=0, $$

where $\langle\cdot,\cdot\rangle$ denotes the Euclidean inner product. $\mathcal{K}=\mathcal{K}^{n_{1}}\times\cdots\times\mathcal{K}^{n_{m}}$ with $m, n_{1},\ldots, n_{m}\geq1$ and $n_{1}+\cdots+n_{m}=n$. $F: R^{n}\times R^{n}\times R^{l}\rightarrow R^{n}\times R^{l}$ is a continuously differentiable mapping. Especially, if $F(x,y,z)=f(x)-y$ with a continuously differentiable function $f: R^{n}\rightarrow R^{n}$ and $\mathcal{K}=\mathcal{K}^{n}$, then we can rewrite SOCCP as follows: Find $(x,y)\in R^{n}\times R^{n}$ such that

$$ x\in\mathcal{K}^{n}, \qquad y\in\mathcal{K}^{n}, \qquad \langle x,y\rangle =0, \qquad y=f(x). $$

If $n_{i}=1$ for all *i*, $l=0$, and the mapping *F* has the form

$$ F(x,y,z)=F_{0}(x)-y $$

for some $F_{0}: R^{n}\rightarrow R^{n}$, the SOCCP become

$$ \bigl\langle x,F_{0}(x)\bigr\rangle =0, \qquad x\geq0,\qquad F_{0}(x)\geq0, $$

which are the well-known nonlinear complementarity problems (NCP).

In fact, many engineering and practical problems, such as the three-dimensional frictional contact problems [1] and the robust Nash equilibria [2], can be changed into SOCCP directly. In addition, as the special case of the SOCCP, some basic theory, effective algorithms, and important applications of complementarity problems in engineering, economics have been developed. However, in practice, several types of uncertain data such as weather, supply, demand, cost, etc. may be involved in SOCCP. The stochastic problems are aimed at a practical treatment of such problems under uncertainty. It is well known that stochastic second-order-cone complementarity problems (SSOCCP) are more complicated than SOCCP and they have found applications in more fields. Therefore, it is meaningful and interesting to study the general SSOCCP.

In this paper, we consider the following SSOCCP: Find vectors $x, y\in R^{n}$ and $z\in R^{l}$ satisfying

1.1 $$ \langle x,y\rangle=0, \qquad x\in\mathcal{K},\qquad y\in \mathcal{K},\qquad F(x,y,z,\xi)=0,\quad \mbox{a.s.}, $$

where $\langle\cdot,\cdot\rangle$ denotes the Euclidean inner product. $\xi\in\Omega$ is a stochastic variable and Ω is the underlying sample space. $F: R^{n}\times R^{n}\times R^{l}\times\Omega \rightarrow R^{n}\times R^{l}$ is a continuously differentiable mapping, and a.s. is the abbreviation for “almost surely” under the given probability measure. Especially, if $F(x,y,z,\xi)=f(x,\xi)-y$ with a continuously differentiable function $f: R^{n}\times\Omega\rightarrow R^{n}$ and $\mathcal{K}=\mathcal{K}^{n}$, then we can rewrite SSOCCP as follows: Find $(x,y)\in R^{n}\times R^{n}$ such that

1.2 $$ x\in\mathcal{K}^{n},\qquad y\in\mathcal{K}^{n}, \qquad \langle x,y\rangle =0, \qquad y=f(x,\xi),\quad \mbox{a.s.} $$

If $n_{i}=1$ for all *i*, $l=0$, and the mapping *F* has the form

$$ F(x,y,z,\xi)=F_{0}(x,\xi)-y $$

for some $F_{0}: R^{n}\times\Omega\rightarrow R^{n}$, the SSOCCP become

1.3 $$ \bigl\langle x,F_{0}(x,\xi)\bigr\rangle =0, \qquad x \geq0, \qquad F_{0}(x,\xi)\geq 0, \quad \mbox{a.s.}, $$

which are the well-known stochastic nonlinear complementarity problems (SNCP). Unless otherwise specified, in the following analysis we assume that $\mathcal{K}=\mathcal{K}^{n}$. This, however, does not lose any generality because our analysis can be easily extended to the general case.

Note that problem (1.3) may not have a solution in general. There have been proposed three ways to deal with (1.3). One way was suggested by Gürkan et al. [3], who used an expectation of $F_{0}$ instead of $F_{0}$ for giving a simple nonlinear complementarity problems reformulation. Another way was presented by Chen and Fukushima [4], who made use of the so-called NCP function to present the expected residual minimization formulation for SNCP. The last was proposed by Lin and Fukushima [5]. They formulated SNCP as a special here-and-now model of stochastic mathematical program with equilibrium constraints. Moreover, Luo and Wang [6] presented the ERM and CVaR reformulation for solving stochastic generalized complementarity problem.

Motivated by the above work, we will propose a reformulation of SSOCCP for giving reasonable solutions of SSOCCP. In the rest of this paper, we assume that Ω is a nonempty compact set. We will often write $x=(x_{1},x_{2})$ for $(x_{1}^{T},x_{2}^{T})^{T}$ and $(x,y,z)$ for $(x^{T},y^{T},z^{T})^{T}$ for simplicity. In addition, for any $x=(x_{1},x_{2})\in R\times R^{n-1}$ and $y=(y_{1},y_{2})\in R\times R^{n-1}$, we define their Jordan product as $x\circ y=(x^{T}y, y_{1}x_{2}+x_{1}y_{2})$. We will write $x^{2}$ to mean $x\circ x$ and write $x+y$ to mean the usual componentwise addition of vectors. Moreover, if $x\in\mathcal{K}^{n}$, then there exists a unique vector in $\mathcal{K}^{n}$, which we denote by $x^{1/2}$ such that $(x^{1/2})^{2}=x^{1/2}\circ x^{1/2}=x$. $F(\cdot,\cdot,\cdot,\xi)$ is twice continuous differentiable with respect to $(x,y,z)$ and $F(x,y,z,\cdot)$ is continuous differentiable with respect to $\xi\in \Omega$. For each $t=1,\ldots, n+l$, $\nabla_{(x,y,z)}F_{t}(x,y,z,\xi )$ denotes the gradient of $F_{t}$ on $(x,y,z)$, $\nabla ^{2}_{(x,y,z)}F_{t}(x,y,z,\xi)$ denotes the Hessian matrix of $F_{t}$ on $(x,y,z)$, and $\frac{\partial F_{t}}{\partial x_{j}}$ denotes a partial derivative on $x_{j}$. $\nabla_{(x,y,z)}F(x,y,z,\xi)=[\nabla _{(x,y,z)}F_{1}(x,y,z,\xi),\ldots,\nabla_{(x,y,z)}F_{n+l}(x,y,z,\xi )]^{T}$. For an $m\times n$ matrix *A*, $\|A\|_{\mathcal{F}}:=\sqrt{\sum_{i=1}^{m}\sum_{j=1}^{n}|a_{ij}|^{2}}$ denotes its Frobenius norm. Moreover, *I* and *O* denote the identity matrix and null matrix with suitable dimensions, respectively.

The remainder of the paper is organized as follows. In Sect. 2, some preliminaries are given and a smoothing sample average approximation problem is presented. The convergence analysis of global solution and weak stationary point of this approximation problem are established in Sect. 3 and Sect. 4, respectively. Moreover, the conclusions are given in Sect. 5.

## Deterministic reformulation and its approximation problems

Because of the existence of a random element *ξ*, we cannot generally expect that there exists a vector $(x,y,z)$ satisfying (1.1) or (1.2) for almost all $\xi\in\Omega$, that is, both (1.1) and (1.2) may not have a solution in general. Therefore, an important issue in the study of SSOCCP is to present an appropriate deterministic formulation of the considered problem. Before giving the reformulation, we first introduce some related functions and their properties.

If $\langle x,y\rangle=0$, $x\in\mathcal{K}^{n}$, $y\in \mathcal{K}^{n} \Longleftrightarrow \phi(x,y)=0$. We call mapping $\phi:R^{n}\times R^{n}\rightarrow R^{n}$ an SOC complementarity function associated with $\mathcal{K}^{n}$. One popular SOC complementarity function which is called by natural residual function was presented by Fukushima et al. in [7]. This function associated with $\mathcal{K}^{n}$ is defined as

$$ \phi_{\mathrm{NR}}(x,y)=x-[x-y]_{+}, $$

where $[x]_{+}$ denotes the projection of *x* onto the second-order-cone $\mathcal{K}^{n}$, that is, $[x]_{+}=\operatorname{argmin}_{{x'}\in\mathcal {K}^{n}}\|x'-x\|$. For simplicity, set $\phi_{0}(x,y):=\phi_{\mathrm{NR}}(x,y)$ in this paper. Note that this function is nondifferentiable but strongly semismooth.

In reference [8], Chen et al. presented a continuously differentiable smoothing function $\phi_{\mu}: R^{n}\times R^{n}\rightarrow R^{n}$ of $\phi_{0}(x,y)$, parameterized by $\mu >0$, such that the pointwise limit $\lim_{\mu\rightarrow0^{+}}\phi _{\mu}(x,y)=\phi_{0}(x,y)$. For any $x=(x_{1},x_{2}),y=(y_{1},y_{2})\in R\times R^{n-1}$,

$$ \phi_{\mu}(x,y)=\frac{1}{2} \bigl[(x+y)- \bigl((x-y)^{2}+4\mu^{2}e \bigr)^{1/2} \bigr], $$

where $\mu>0$ is a smoothing parameter and $e=(1,0,\ldots,0)^{T}\in R^{n}$. Especially, by Proposition 6.2 of [9], we know that $\phi_{\mu}(x,y)$ is globally Lipschitz continuous, which means that there exists a positive constant $\nu>0$ such that

$$ \bigl\Vert \phi_{\mu}(x,y)-\phi_{0}(x,y) \bigr\Vert \leq\nu\mu. $$

Motivated by the work of Chen and Fukushima [4] for the special case of SSOCCP, we propose the following deterministic formulation for SSOCCP, called the ERM problem below, in which we try to find a vector $(x,y,z)\in R^{n}\times R^{n}\times R^{l}$ that minimizes an expected residual for $\phi_{0}$ and $F(x,y,z,\xi)$, that is,

2.1 $$ \min_{(x,y,z)} \theta(x,y,z):=\mathbb{E} \bigl[ \bigl\Vert F(x,y,z,\xi) \bigr\Vert ^{2}+ \bigl\Vert \phi_{0}(x,y) \bigr\Vert ^{2} \bigr]. $$

Here, $\mathbb{E}$ stands for the expectation with respect to the random variable $\xi\in\Omega$.

It is well known that $\phi_{0}$ is not differentiable with respect to $(x,y)$. Hence, for solving ERM model, we consider the following smoothing approximation problem of (2.1):

2.2 $$ \min_{(x,y,z)} \theta_{\mu}(x,y,z):= \mathbb{E} \bigl[ \bigl\Vert F(x,y,z,\xi) \bigr\Vert ^{2}+ \bigl\Vert \phi_{\mu}(x,y) \bigr\Vert ^{2} \bigr]. $$

Since an expectation function is generally difficult to evaluate exactly, we employ a sample average approximation (SAA) method for numerical integration to solve (2.2). Thus, by taking independent identically distributed (i.i.d.) samples $\xi^{1},\ldots, \xi^{N_{k}}$, one may construct, for each *k*, a smoothing sample average approximation to problem (2.2) as follows:

2.3 $$ \min_{(x,y,z)} \theta_{\mu}^{k}(x,y,z):= \frac{1}{N_{k}}\sum_{\xi^{i}\in \Omega_{k}} \bigl\Vert F\bigl(x,y,z, \xi^{i}\bigr) \bigr\Vert ^{2}+ \bigl\Vert \phi_{\mu}(x,y) \bigr\Vert ^{2}, $$

where $\Omega_{k}:=\{\xi^{i}| i=1,\ldots, N_{k}\}$ is a set of observations generated by a sample average approximation method such that $\Omega_{k}\subset\Omega$ and $N_{k}\rightarrow\infty$ as $k\rightarrow+\infty$.

For the sample average approximation method, the strong law of large numbers guarantees that the following result holds with probability one (abbreviated by “w.p.1.”).

### Lemma 2.1

[10, 11] *Let* $\eta: \Omega\to R$ *be integrable over* Ω. *Then we have*

$$ \lim_{k\rightarrow+\infty}\frac{1}{N_{k}}\sum_{{\xi^{i}}\in\Omega_{k}} \eta\bigl(\xi^{i}\bigr)=\mathbb{E}\bigl[ \eta(\xi)\bigr], \quad \textit{w.p.}1. $$

It is generally known that the boundedness of an iteration sequence is the basic requirement in the iteration methods for solving various optimization problems. To ensure the boundedness of the iteration sequence of problem (2.1), it is essential to take the level sets of $\theta(x,y,z)$ into account. And it is worth noting that if $\theta(x,y,z)$ is coercive, i.e., $\lim_{\|x\|\rightarrow \infty}{\theta(x,y,z)}=+\infty$, then the level sets of $\theta (x,y,z)$ are bounded. Next, we study the coerciveness of $\theta (x,y,z)$ under appropriate conditions.

### Theorem 2.1

*If the sequence* $\{(x^{k},y^{k})\}\subseteq R^{n}\times R^{n}$ *satisfies* $\liminf_{k\rightarrow+\infty}\lambda_{1}(x^{k})=-\infty$ *or* $\liminf_{k\rightarrow+\infty}\lambda_{1}(y^{k})=-\infty$, *then* $\|\theta(x^{k},y^{k},z^{k})\|\rightarrow+\infty$ *as* $k\rightarrow +\infty$. *Here*, $\lambda_{1}(x)$ *is the spectral value of* $x=(x_{1}, x_{2})\in R\times R^{n-1}$, *given by* $\lambda_{1}(x)=x_{1}-\|x_{2}\|$.

### Proof

It is easy to obtain the conclusion from Lemma 3.5 of [9]. In addition, more details about Jordan algebra associated with SOCCP also can be seen in [9]. □

## Convergence of global optimal solution

In this section, we will show that the global solution sequence of smoothing approximation problem (2.2) converges to the global solution of the true ERM problem (2.1). Then we will consider that the global solution sequence of smoothing SAA approximation problem (2.3) converges to the global solution of the so-called true problem (2.1) when the smoothing parameter tends to 0^+^ as sample size increases to +∞.

From now on, we denote by $\bar{S}(\mu)$ the set of global optimal solutions of problem (2.2).

### Theorem 3.1

*Suppose that for each* *μ*, $(x(\mu),y(\mu),z(\mu))$ *is a global optimal solution of problem* (2.2) *and* $\lim_{\mu\to0^{+}} x(\mu)=\hat{x}$, $\lim_{\mu\to0^{+}} y(\mu )=\hat{y}$, $\lim_{\mu\to0^{+}} z(\mu)=\hat{z}$. *Then we have* $(\hat{x},\hat{y},\hat{z})$ *is a global optimal solution of problem* (2.1).

### Proof

Let *D* be a compact convex set containing the sequence $\{(x(\mu), y(\mu), z(\mu))\}$. By the continuous differentiability of *F* on the compact set $D\times\Omega$ and Theorem 16.8 of [12], we have

3.1 $$\begin{aligned} \lim_{\mu\rightarrow0^{+}}\mathbb{E} \bigl[ \bigl\Vert F\bigl(x(\mu),y( \mu),z(\mu ),\xi\bigr) \bigr\Vert ^{2} \bigr] =&\mathbb{E} \Bigl[\lim _{\mu\rightarrow0^{+}} \bigl\Vert F\bigl(x(\mu),y(\mu),z(\mu),\xi\bigr) \bigr\Vert ^{2} \Bigr] \\ =&\mathbb{E} \bigl[ \bigl\Vert F(\hat {x},\hat{y},\hat{z},\xi) \bigr\Vert ^{2} \bigr]. \end{aligned}$$

Noting that

$$\begin{aligned}& \bigl\Vert \phi_{0}\bigl(x(\mu),y(\mu)\bigr)-\phi_{0}( \hat{x},\hat{y}) \bigr\Vert \\& \quad \leq \bigl\Vert x(\mu)-\hat{x} \bigr\Vert + \bigl\Vert \bigl[x( \mu)-y(\mu)\bigr]_{+}-[\hat{x}-\hat {y}]_{+} \bigr\Vert \\& \quad \leq 2 \bigl\Vert x(\mu)-\hat{x} \bigr\Vert + \bigl\Vert y(\mu)- \hat{y} \bigr\Vert , \end{aligned}$$

where the first inequality follows from the definition of $\phi_{0}$, the second inequality follows from the nonexpansive property of project. Then we have

3.2 $$ \lim_{\mu\rightarrow0^{+}} \bigl\Vert \phi_{0} \bigl(x(\mu),y(\mu)\bigr) \bigr\Vert = \bigl\Vert \phi _{0}( \hat{x},\hat{y}) \bigr\Vert . $$

It then follows from (3.2) and the fact $\phi_{0}=\lim_{\mu \rightarrow0^{+}}\phi_{\mu}$ that

3.3 $$\begin{aligned}& \bigl\Vert \phi_{\mu}\bigl(x(\mu),y(\mu)\bigr) \bigr\Vert \\& \quad \leq \bigl\Vert \phi_{\mu}\bigl(x(\mu),y(\mu)\bigr)- \phi_{0}\bigl(x(\mu),y(\mu)\bigr) \bigr\Vert + \bigl\Vert \phi _{0}\bigl(x(\mu),y(\mu)\bigr) \bigr\Vert \\& \quad \xrightarrow{\mu\rightarrow0^{+}} \bigl\Vert \phi_{0}(\hat{x}, \hat{y}) \bigr\Vert . \end{aligned}$$

Since $(x(\mu),y(\mu),z(\mu))$ is a global optimal solution of problem (2.2), there holds

3.4 $$ \theta_{\mu}\bigl(x(\mu),y(\mu),z(\mu)\bigr)\leq \theta_{\mu}(x,y,z),\quad \forall (x,y,z)\in R^{n}\times R^{n}\times R^{l}. $$

Letting $\mu\to0^{+}$ in (3.4), we get from (3.1), (3.3), and the fact $\phi_{0}=\lim_{\mu \rightarrow0^{+}}\phi_{\mu}$ that

$$ \theta(\hat{x},\hat{y},\hat{z})\leq\theta(x,y,z), \quad \forall (x,y,z)\in R^{n}\times R^{n}\times R^{l}, $$

which indicates $(\hat{x},\hat{y},\hat{z})$ is a global optimal solution of problem (2.1). □

Let *μ* vary as *k* increases, that is, let $\mu=\mu_{k}\rightarrow 0^{+}$ as $k\rightarrow+\infty$. In the following, we discuss the convergence for this case.

### Theorem 3.2

*Suppose that for each* *k*, $(x^{k}(\mu_{k}),y^{k}(\mu_{k}),z^{k}(\mu_{k}))$ *is a global optimal solution of problem* (2.3) *and* $\lim_{k\to+\infty} x^{k}(\mu_{k})=\bar{x}$, $\lim_{k\to+\infty} y^{k}(\mu_{k})=\bar{y}$, $\lim_{k\to+\infty} z^{k}(\mu_{k})=\bar{z}$, *w*.*p*.1. *Then we have* $(\bar{x},\bar{y},\bar{z})$ *is a global optimal solution of problem* (2.1) *w*.*p*.1.

### Proof

Let *A* be a compact convex set containing the sequence $\{(x^{k}(\mu_{k}), y^{k}(\mu_{k}), z^{k}(\mu_{k}))\}$. By the continuous differentiability of *F* on the compact set $A\times\Omega$, there exist constants $C_{1}>0$, $C_{2}>0$ such that

3.5 $$\begin{aligned}& \bigl\Vert F(x,y,z,\xi) \bigr\Vert \le C_{1}, \end{aligned}$$

3.6 $$\begin{aligned}& \bigl\Vert \nabla F(x,y,z,\xi) \bigr\Vert _{\mathcal{F}}\le C_{2},\quad \forall (x,y,z,\xi)\in A\times\Omega. \end{aligned}$$

Moreover, we have from the mean-value theorem that, for each *k* and each $\xi^{i}$, there exist $\gamma^{ki}_{x}(\mu_{k})=\alpha_{ki}x^{k}(\mu_{k})+(1-\alpha_{ki}) \bar {x}$, $\gamma^{ki}_{y}(\mu_{k})=\alpha_{ki}y^{k}(\mu_{k})+(1-\alpha_{ki}) \bar{y}$, $\gamma^{ki}_{z}(\mu_{k})=\alpha_{ki}z^{k}(\mu_{k})+(1-\alpha _{ki}) \bar{z}$, and $(\gamma^{ki}_{x}(\mu_{k}), \gamma^{ki}_{y}(\mu _{k}), \gamma^{ki}_{z}(\mu_{k}))\in A$ with $\alpha_{ki}\in[0,1]$ such that

3.7 $$\begin{aligned}& F_{t}\bigl(x^{k}(\mu_{k}),y^{k}( \mu_{k}),z^{k}(\mu_{k}),\xi^{i} \bigr)-F_{t}\bigl(\bar{x},\bar {y},\bar{z},\xi^{i}\bigr) \\& \quad = \nabla_{(x,y,z)}F_{t}\bigl(\gamma^{ki}_{x}( \mu_{k}), \gamma^{ki}_{y}(\mu_{k}), \gamma^{ki}_{z}(\mu_{k}), \xi^{i} \bigr)^{T}\bigl(x^{k}(\mu_{k})-\bar{x},y^{k}( \mu_{k})-\bar {y},z^{k}(\mu_{k})-\bar{z} \bigr). \end{aligned}$$

Then we have

3.8 $$\begin{aligned}& \bigl\vert F_{t}^{2}\bigl(x^{k}( \mu_{k}),y^{k}(\mu_{k}),z^{k}( \mu_{k}),\xi^{i}\bigr)-F_{t}^{2}\bigl( \bar {x},\bar{y},\bar{z},\xi^{i}\bigr) \bigr\vert \\& \quad \leq \bigl\vert F_{t}\bigl(x^{k}( \mu_{k}),y^{k}(\mu_{k}),z^{k}( \mu_{k}),\xi^{i}\bigr)+F_{t}\bigl(\bar {x},\bar{y}, \bar{z},\xi^{i}\bigr) \bigr\vert \\& \qquad {}\cdot \bigl\vert F_{t}\bigl(x^{k}( \mu_{k}),y^{k}(\mu_{k}),z^{k}( \mu_{k}),\xi^{i}\bigr)-F_{t}\bigl(\bar{x},\bar {y}, \bar{z},\xi^{i}\bigr) \bigr\vert \\& \quad \leq 2C_{1} \bigl\Vert \nabla_{(x,y,z)}F_{t} \bigl(\gamma^{ki}_{x}(\mu_{k}), \gamma ^{ki}_{y}(\mu_{k}), \gamma^{ki}_{z}( \mu_{k}), \xi^{i}\bigr) \bigr\Vert _{\mathcal{F}} \\& \qquad {}\cdot \bigl\Vert \bigl(x^{k}(\mu_{k})-\bar{x},y^{k}( \mu_{k})-\bar{y},z^{k}(\mu_{k})-\bar{z}\bigr) \bigr\Vert \\& \quad \leq 2C_{1}C_{2}\bigl( \bigl\Vert x^{k}( \mu_{k})-\bar{x} \bigr\Vert + \bigl\Vert y^{k}( \mu_{k})-\bar{y} \bigr\Vert + \bigl\Vert z^{k}( \mu_{k})-\bar{z} \bigr\Vert \bigr), \end{aligned}$$

where the second inequality follows from (3.5), the third inequality follows from (3.6), the definition of $\|\cdot\| _{\mathcal{F}}$, and the fact that $\sqrt{A+B}\leq\sqrt{A}+\sqrt{B}$ with $A\geq0$, $B\geq0$.

It then follows from Lemma 2.1 and (3.8) that

3.9 $$\begin{aligned}& \biggl\vert \frac{1}{N_{k}}\sum_{\xi^{i}\in\Omega_{k}} \bigl\Vert F\bigl(x^{k}(\mu _{k}),y^{k}( \mu_{k}),z^{k}(\mu_{k}),\xi^{i}\bigr) \bigr\Vert ^{2}-\mathbb{E} \bigl[ \bigl\Vert F(\bar {x},\bar{y}, \bar{z},\xi) \bigr\Vert ^{2} \bigr] \biggr\vert \\& \quad \leq \frac{1}{N_{k}}\sum_{\xi^{i}\in\Omega_{k}}\sum _{t=1}^{n+l} \bigl\vert F_{t}^{2} \bigl(x^{k}(\mu_{k}),y^{k}(\mu_{k}),z^{k}( \mu_{k}),\xi^{i}\bigr)-F_{t}^{2}\bigl( \bar{x},\bar {y},\bar{z},\xi^{i}\bigr) \bigr\vert \\& \qquad {} + \biggl\vert \frac{1}{N_{k}}\sum_{\xi^{i}\in\Omega_{k}} \bigl\Vert F\bigl(\bar{x},\bar {y},\bar{z},\xi^{i}\bigr) \bigr\Vert ^{2}-\mathbb{E} \bigl[ \bigl\Vert F(\bar{x},\bar{y},\bar {z},\xi) \bigr\Vert ^{2} \bigr] \biggr\vert \\& \quad \leq 2C_{1}C_{2}(n+l)\frac{1}{N_{k}}\sum _{\xi^{i}\in\Omega_{k}} \bigl( \bigl\Vert \bigl(x^{k}( \mu_{k})-\bar{x} \bigr\Vert + \bigl\Vert y^{k}( \mu_{k})-\bar{y} \bigr\Vert + \bigl\Vert z^{k}( \mu_{k})-\bar {z}\bigr) \bigr\Vert \bigr) \\& \qquad {} + \biggl\vert \frac{1}{N_{k}}\sum_{\xi^{i}\in\Omega_{k}} \bigl\Vert F\bigl(\bar{x},\bar {y},\bar{z},\xi^{i}\bigr) \bigr\Vert ^{2}-\mathbb{E} \bigl[ \bigl\Vert F(\bar{x},\bar{y},\bar {z},\xi) \bigr\Vert ^{2} \bigr] \biggr\vert \\& \quad \xrightarrow{k\rightarrow+\infty} 0,\quad \mbox{w.p.}1. \end{aligned}$$

Similar to obtaining (3.3), we have

3.10 $$ \lim_{k\rightarrow+\infty} \bigl\Vert \phi_{\mu_{k}} \bigl(x^{k}(\mu_{k}),y^{k}(\mu _{k})\bigr) \bigr\Vert ^{2} = \bigl\Vert \phi_{0}(\bar{x},\bar{y}) \bigr\Vert ^{2}. $$

Since

$$\begin{aligned}& \bigl\vert \theta_{\mu_{k}}^{k}\bigl(x^{k}( \mu_{k}),y^{k}(\mu_{k}),z^{k}( \mu_{k})\bigr)-\theta (\bar{x},\bar{y},\bar{z}) \bigr\vert \\& \quad \leq \biggl\vert \frac{1}{N_{k}}\sum_{\xi^{i}\in\Omega_{k}} \bigl\Vert F\bigl(x^{k}(\mu _{k}),y^{k}( \mu_{k}),z^{k}(\mu_{k}),\xi^{i}\bigr) \bigr\Vert ^{2}-\mathbb{E} \bigl[ \bigl\Vert F(\bar {x},\bar{y}, \bar{z},\xi) \bigr\Vert ^{2} \bigr] \biggr\vert \\& \qquad {}+ \bigl\vert \bigl\Vert \phi_{\mu_{k}}\bigl(x^{k}( \mu_{k}),y^{k}(\mu_{k})\bigr) \bigr\Vert ^{2}- \bigl\Vert \phi_{0}(\bar {x},\bar{y}) \bigr\Vert ^{2} \bigr\vert , \end{aligned}$$

we have from (3.9) and (3.10) that

3.11 $$ \lim_{k\to+\infty}\theta_{\mu_{k}}^{k} \bigl(x^{k}(\mu_{k}),y^{k}(\mu_{k}),z^{k}( \mu _{k})\bigr)=\theta(\bar{x},\bar{y},\bar{z}), \quad \mbox{ w.p.}1. $$

On the other hand, since $(x^{k}(\mu_{k}),y^{k}(\mu_{k}),z^{k}(\mu_{k}))$ is a global optimal solution of problem (2.3), there holds

3.12 $$ \theta_{\mu_{k}}^{k}\bigl(x^{k}( \mu_{k}),y^{k}(\mu_{k}),z^{k}( \mu_{k})\bigr)\leq\theta _{\mu_{k}}^{k}(x,y,z), \quad \forall (x,y,z)\in R^{n}\times R^{n}\times R^{l}. $$

Letting $k\to+\infty$ in (3.12), we get from (3.11), Lemma 2.1, and the limit $\phi _{0}=\lim_{\mu\rightarrow0^{+}}\phi_{\mu}$ that

$$ \theta(\bar{x},\bar{y},\bar{z})\leq\theta(x,y,z), \quad \forall (x,y,z)\in R^{n}\times R^{n}\times R^{l}, $$

which indicates $(\bar{x},\bar{y},\bar{z})$ is a global optimal solution of problem (2.1). □

## Convergence of weak stationary point

We first give some definitions associated with a weak stationary point of problem (2.1), then we show that the sequence of weak stationary point for (2.2) converges to a weak stationary point for (2.1) as $\mu\rightarrow0^{+}$. Then we will consider the convergence of the stationary point of (2.3) while $\mu=\mu_{k}\rightarrow0^{+}$ as $k\rightarrow+\infty$.

### Definition 4.1

Let $g: R^{n}\rightarrow R$ be locally Lipschitz continuous. The Clarke generalized gradient of *g* at $x_{0}$ is defined as

4.1 $$ \partial_{x}g(x_{0}):=\operatorname{Co}\Bigl\{ \lim _{x'\rightarrow x_{0}}\nabla_{x}g\bigl(x'\bigr)\Bigr\} , $$

where $\operatorname{Co}\{X\}$ denotes the convex hull of a set *X*.

### Definition 4.2

A point $(\tilde{x},\tilde{y},\tilde{z})$ satisfying the following equation is called a weak stationary point of (2.1):

4.2 $$ 0\in2\mathbb{E}\bigl[\nabla_{(x,y,z)}F(\tilde{x},\tilde{y}, \tilde {z},\xi)^{T}F(\tilde{x},\tilde{y},\tilde{z},\xi)\bigr]+\partial _{(x,y,z)} \bigl\Vert \phi_{0}(\tilde{x},\tilde{y}) \bigr\Vert ^{2}, $$

where $\partial_{(x,y,z)}\|\phi_{0}(\tilde{x},\tilde{y})\|^{2}$ denotes the Clarke generalized gradient of $\|\phi_{0}(\tilde{x},\tilde{y})\| ^{2}$ at $(\tilde{x},\tilde{y},\tilde{z})$.

### Definition 4.3

For each fixed *μ*, a point $(x(\mu),y(\mu),z(\mu))$ satisfying the following equation is called a weak stationary point of (2.2):

4.3 $$\begin{aligned}& \mathbb{E}\bigl[\nabla_{(x,y,z)}F\bigl(x(\mu),y(\mu),z(\mu),\xi \bigr)^{T}F\bigl(x(\mu ),y(\mu),z(\mu),\xi\bigr)\bigr] \\& \quad {} + \bigl(\nabla_{(x,y)}\phi_{\mu}\bigl(x(\mu),y(\mu) \bigr)^{T}\phi_{\mu}\bigl(x(\mu),y(\mu)\bigr),\mathbf{0} \bigr)=0, \end{aligned}$$

where $\mathbf{0}\in R^{l}$ is a zero vector.

### Definition 4.4

For each fixed *μ* and *k*, a point $(x^{k}(\mu),y^{k}(\mu),z^{k}(\mu ))$ satisfying the following equation is called a stationary point of (2.3):

4.4 $$\begin{aligned}& \frac{1}{N_{k}}\sum_{\xi^{i}\in\Omega_{k}}\bigl[ \nabla_{(x,y,z)}F\bigl(x^{k}(\mu ),y^{k}( \mu),z^{k}(\mu),\xi^{i}\bigr)^{T}F \bigl(x^{k}(\mu),y^{k}(\mu),z^{k}(\mu),\xi ^{i}\bigr)\bigr] \\& \quad {} + \bigl(\nabla _{(x,y)}\phi_{\mu}\bigl(x^{k}( \mu),y^{k}(\mu)\bigr)^{T}\phi_{\mu}\bigl(x^{k}(\mu),y^{k}(\mu )\bigr),\mathbf{0} \bigr)=0. \end{aligned}$$

Especially, more details about weak stationary points can be seen in [13].

### Theorem 4.1

*Let* $(x(\mu),y(\mu),z(\mu))$ *be a weak stationary point of* (2.2) *for each* *μ* *and* $\lim_{\mu\to0^{+}} x(\mu)=\tilde{x}$, $\lim_{\mu\to0^{+}} y(\mu )=\tilde{y}$, $\lim_{\mu\to0^{+}} z(\mu)=\tilde{z}$. *Suppose that* $\|\phi_{\mu}(x,y)\|^{2}$ *satisfies gradient consistency*. *Then* $(\tilde {x},\tilde{y},\tilde{z})$ *is a weak stationary point of problem* (2.1).

### Proof

Let **D** be a compact convex set containing the sequence $\{(x(\mu), y(\mu), z(\mu))\}$. By the twice continuous differentiability of *F* on the compact set $\mathbf{D}\times\Omega$ and Theorem 16.8 of [12], we have

4.5 $$\begin{aligned}& \lim_{\mu\rightarrow0^{+}}\mathbb{E} \bigl[\nabla _{(x,y,z)}F\bigl(x( \mu),y(\mu),z(\mu),\xi\bigr)^{T}F\bigl(x(\mu),y(\mu),z(\mu ),\xi\bigr) \bigr] \\& \quad = \mathbb{E} \Bigl[\lim_{\mu\rightarrow0^{+}}\nabla _{(x,y,z)}F \bigl(x(\mu),y(\mu),z(\mu),\xi\bigr)^{T}F\bigl(x(\mu),y(\mu),z(\mu ),\xi \bigr) \Bigr] \\& \quad = \mathbb{E} \bigl[\nabla_{(x,y,z)}F(\tilde{x},\tilde{y},\tilde {z}, \xi)^{T}F(\tilde{x},\tilde{y},\tilde{z},\xi) \bigr]. \end{aligned}$$

Since $\nabla_{(x,y)}\phi_{\mu}(x,y)$ is continuously on **D**, there exists $C_{3}>0$ such that

4.6 $$ \bigl\Vert \nabla_{(x,y)}\phi_{\mu}(x,y) \bigr\Vert \leq C_{3}. $$

Noting (4.6) and the gradient consistency of $\|\phi_{\mu}(x,y)\|^{2}$, we know that the conditions of Theorem 3.1 in [13] hold. Taking a limit in (4.3), by (4.5), and the results of Theorem 3.1 in [13], we obtain (4.2) immediately. That is, $(\tilde{x},\tilde{y},\tilde{z})$ is a weak stationary point of problem (2.1). □

### Remark 4.1

The condition $\|\phi_{\mu}(x,y)\|^{2}$ satisfying gradient consistency has been proved in [14]. In fact, the smoothing function $\phi_{\mu}$ used in this paper is a special form of smoothing function considered in [14].

### Lemma 4.1

*Suppose that* $\lim_{k\rightarrow+\infty}x^{k}(\mu_{k})=\tilde{x}$, $\lim_{k\rightarrow+\infty}y^{k}(\mu_{k})=\tilde{y}$, $\lim_{k\rightarrow+\infty}z^{k}(\mu_{k})=\tilde{z}$, *w*.*p*.1. *Then we have that the following limit holds with probability one*:

$$\begin{aligned}& \lim_{k\rightarrow+\infty}\frac{1}{N_{k}}\sum_{\xi^{i}\in\Omega _{k}} \sum_{t=1}^{n+l}F_{t} \bigl(x^{k}(\mu_{k}),y^{k}(\mu_{k}),z^{k}( \mu_{k}),\xi ^{i}\bigr)\cdot\frac{\partial F_{t}(x^{k}(\mu_{k}),y^{k}(\mu_{k}),z^{k}(\mu_{k}),\xi ^{i})}{\partial x_{j}} \\& \quad =\mathbb{E} \Biggl[\sum_{t=1}^{n+l}F_{t}( \tilde{x},\tilde{y},\tilde {z},\xi)\cdot\frac{\partial F_{t}(\tilde{x},\tilde{y},\tilde{z},\xi )}{\partial x_{j}} \Biggr], \quad j=1, \ldots,n. \end{aligned}$$

### Proof

Let **A** be a compact convex set containing the sequence $\{(x^{k}(\mu_{k}), y^{k}(\mu_{k}), z^{k}(\mu_{k}))\}$. By the twice continuous differentiability of *F* on the compact set **A** and the continuity of *F* with respect to *ξ* on a compact set Ω, there exist constants $C_{4}>0$, $C_{5}>0$, $C_{6}>0$ such that

4.7 $$\begin{aligned}& \bigl\Vert F(x,y,z,\xi) \bigr\Vert \le C_{4}, \end{aligned}$$

4.8 $$\begin{aligned}& \bigl\Vert \nabla_{(x,y,z)} F(x,y,z,\xi) \bigr\Vert _{\mathcal{F}} \le C_{5}, \end{aligned}$$

4.9 $$\begin{aligned}& \bigl\{ \max \bigl\Vert \nabla_{(x,y,z)}^{2}F_{t}(x,y,z, \xi) \bigr\Vert _{\mathcal{F}}, t=1,\ldots,n+l\bigr\} \le C_{6}, \quad \forall (x,y,z,\xi)\in\mathbf{A}\times\Omega. \end{aligned}$$

Similar to obtaining (3.7), taking the twice continuous differentiability of *F* and (4.9) into account, we have

4.10 $$\begin{aligned}& \bigl\vert F_{t}\bigl(x^{k}(\mu_{k}),y^{k}( \mu_{k}),z^{k}(\mu_{k}),\xi^{i} \bigr)-F_{t}\bigl(\tilde {x},\tilde{y},\tilde{z},\xi^{i} \bigr) \bigr\vert \\& \quad \leq C_{5} \bigl\Vert \bigl(x^{k}( \mu_{k})-\tilde{x}, y^{k}(\mu_{k})-\tilde{y}, z^{k}(\mu _{k})-\tilde{z}\bigr) \bigr\Vert , \end{aligned}$$

4.11 $$\begin{aligned}& \biggl\vert \frac{\partial F_{t}(x^{k}(\mu_{k}),y^{k}(\mu_{k}),z^{k}(\mu_{k}),\xi ^{i})}{\partial x_{j}}-\frac{\partial F_{t}(\tilde{x},\tilde{y},\tilde {z},\xi^{i})}{\partial x_{j}} \biggr\vert \\& \quad \leq C_{6} \bigl\Vert \bigl(x^{k}( \mu_{k})-\tilde{x}, y^{k}(\mu_{k})-\tilde{y}, z^{k}(\mu _{k})-\tilde{z}\bigr) \bigr\Vert . \end{aligned}$$

Then, for $j=1,\ldots,n$, we have

$$\begin{aligned}& \Biggl\vert \frac{1}{N_{k}}\sum_{\xi^{i}\in\Omega_{k}}\sum _{t=1}^{n+l} \bigl(F_{t} \bigl(x^{k}(\mu_{k}),y^{k}(\mu_{k}),z^{k}( \mu_{k}),\xi^{i}\bigr)\bigr)\cdot\frac {\partial F_{t}(x^{k}(\mu_{k}),y^{k}(\mu_{k}),z^{k}(\mu_{k}),\xi^{i})}{\partial x_{j}} \\& \qquad {}-\mathbb{E} \Biggl[\sum_{t=1}^{n+l} \bigl(F_{t}(\tilde{x},\tilde{y},\tilde{z},\xi) \bigr)\cdot \frac{\partial F_{t}(\tilde{x},\tilde{y},\tilde{z},\xi )}{\partial x_{j}}\Biggr] \Biggr\vert \\& \quad \leq \frac{1}{N_{k}}\sum_{\xi^{i}\in\Omega_{k}}\sum _{t=1}^{n+l} \bigl\vert F_{t} \bigl(x^{k}(\mu_{k}),y^{k}(\mu_{k}),z^{k}( \mu_{k}),\xi^{i}\bigr)-F_{t}\bigl(\tilde{x},\tilde {y},\tilde{z},\xi^{i}\bigr) \bigr\vert \\& \qquad {}\cdot \biggl\vert \frac {\partial F_{t}(x^{k}(\mu_{k}),y^{k}(\mu_{k}),z^{k}(\mu_{k}),\xi^{i})}{\partial x_{j}} \biggr\vert \\& \qquad {} +\frac{1}{N_{k}}\sum_{\xi^{i}\in\Omega_{k}}\sum _{t=1}^{n+l} \biggl\vert F_{t}\bigl( \tilde{x},\tilde{y},\tilde{z},\xi^{i}\bigr)\cdot\biggl( \frac {\partial F_{t}(x^{k}(\mu_{k}),y^{k}(\mu_{k}),z^{k}(\mu_{k}),\xi^{i})}{\partial x_{j}}-\frac{\partial F_{t}(\tilde{x},\tilde{y},\tilde{z},\xi ^{i})}{\partial x_{j}}\biggr) \biggr\vert \\& \qquad {} + \Biggl\vert \frac{1}{N_{k}}\sum_{\xi^{i}\in\Omega_{k}} \sum_{t=1}^{n+l} \biggl(F_{t}\bigl( \tilde{x},\tilde{y},\tilde{z},\xi^{i}\bigr)\cdot\frac{\partial F_{t}(\tilde{x},\tilde{y},\tilde{z},\xi^{i})}{\partial x_{j}} \biggr) \\& \qquad {}-\mathbb{E}\Biggl[\sum_{t=1}^{n+l}F_{t}( \tilde{x},\tilde{y},\tilde {z},\xi)\cdot\frac{\partial F_{t}(\tilde{x},\tilde{y},\tilde{z},\xi )}{\partial x_{j}}\Biggr] \Biggr\vert \\& \quad \leq (C_{5}+C_{6}) \bigl\Vert \bigl(x^{k}(\mu_{k})-\tilde{x}, y^{k}( \mu_{k})-\tilde{y}, z^{k}(\mu_{k})-\tilde{z}\bigr) \bigr\Vert \\& \qquad {} \cdot\frac{1}{N_{k}}\sum_{\xi^{i}\in\Omega_{k}}\sum _{t=1}^{n+l} \biggl( \biggl\vert \frac{\partial F_{t}(x^{k}(\mu_{k}),y^{k}(\mu_{k}),z^{k}(\mu_{k}),\xi ^{i})}{\partial x_{j}} \biggr\vert + \bigl\vert F_{t}\bigl(\tilde{x}, \tilde{y},\tilde{z},\xi^{i}\bigr) \bigr\vert \biggr) \\& \qquad {} + \Biggl\vert \frac{1}{N_{k}}\sum_{\xi^{i}\in\Omega_{k}} \sum_{t=1}^{n+l} \biggl(F_{t}\bigl( \tilde{x},\tilde{y},\tilde{z},\xi^{i}\bigr)\cdot\frac{\partial F_{t}(\tilde{x},\tilde{y},\tilde{z},\xi^{i})}{\partial x_{j}} \biggr) \\& \qquad {}-\mathbb{E}\Biggl[\sum_{t=1}^{n+l}F_{t}( \tilde{x},\tilde{y},\tilde {z},\xi)\cdot\frac{\partial F_{t}(\tilde{x},\tilde{y},\tilde{z},\xi )}{\partial x_{j}}\Biggr] \Biggr\vert \\& \quad \xrightarrow{k\rightarrow+\infty} 0,\quad \mbox{w.p.}1., \end{aligned}$$

where the second inequality follows from (4.8), (4.9), (4.10), (4.11), and the last limit follows from Lemma 2.1, (4.7), and (4.8). This means the conclusion holds. □

### Theorem 4.2

*Let* $(x^{k}(\mu_{k}),y^{k}(\mu_{k}),z^{k}(\mu_{k}))$ *be a stationary point to* (2.3) *for each* *k* *and* $\lim_{k\rightarrow+\infty}x^{k}(\mu_{k})=\tilde{x}$, $\lim_{k\rightarrow+\infty}y^{k}(\mu_{k})=\tilde{y}$, $\lim_{k\rightarrow +\infty}z^{k}(\mu_{k})=\tilde{z}$ *w*.*p*.1. *Then* $(\tilde{x},\tilde {y},\tilde{z})$ *is a weak stationary point of problem* (2.1) *w*.*p*.1.

### Proof

Similar to the proof of Lemma 4.1, we have

4.12 $$\begin{aligned}& \lim_{k\rightarrow+\infty}\frac{1}{N_{k}}\sum_{\xi^{i}\in\Omega _{k}} \sum_{t=1}^{n+l}F_{t} \bigl(x^{k}(\mu_{k}),y^{k}(\mu_{k}),z^{k}( \mu_{k}),\xi ^{i}\bigr)\cdot\frac{\partial F_{t}(x^{k}(\mu_{k}),y^{k}(\mu_{k}),z^{k}(\mu_{k}),\xi ^{i})}{\partial y_{j}} \\& \quad =\mathbb{E} \Biggl[\sum_{t=1}^{n+l}F_{t}( \tilde{x},\tilde{y},\tilde {z},\xi)\cdot\frac{\partial F_{t}(\tilde{x},\tilde{y},\tilde{z},\xi )}{\partial y_{j}} \Biggr],\quad j=1, \ldots,n, \end{aligned}$$

4.13 $$\begin{aligned}& \lim_{k\rightarrow+\infty}\frac{1}{N_{k}}\sum_{\xi^{i}\in\Omega _{k}} \sum_{t=1}^{n+l}F_{t} \bigl(x^{k}(\mu_{k}),y^{k}(\mu_{k}),z^{k}( \mu_{k}),\xi ^{i}\bigr)\cdot\frac{\partial F_{t}(x^{k}(\mu_{k}),y^{k}(\mu_{k}),z^{k}(\mu_{k}),\xi ^{i})}{\partial z_{j}} \\& \quad =\mathbb{E} \Biggl[\sum_{t=1}^{n+l}F_{t}( \tilde{x},\tilde{y},\tilde {z},\xi)\cdot\frac{\partial F_{t}(\tilde{x},\tilde{y},\tilde{z},\xi )}{\partial z_{j}} \Biggr],\quad j=1, \ldots,l. \end{aligned}$$

Noting (4.6) and the gradient consistency of $\|\phi_{\mu}(x,y)\|^{2}$, we know that the conditions of Theorem 4.4 in [13] hold. Taking a limit in (4.4), by Lemma 4.1, (4.12), (4.13), and the results of Theorem 4.4 in [13], we obtain (4.2) immediately. That is, $(\tilde{x},\tilde{y},\tilde{z})$ is a weak stationary point of problem (2.1). □

## Numerical examples

In this section, we use random generator *rand* to generate a random sequence $\{\xi^{1},\ldots,\xi^{N_{k}}\}$ of *ξ* and employ the Matlab unconstrained optimization command *fminunc* to solve the corresponding problems (2.1) and (2.3).

### Example 5.1

Let *ξ* be the uniformly distributed random variable in $(0,1)$, and $F:R^{2}\times R^{2}\times\Omega\rightarrow R^{2}$ is defined as follows:

$$ F(x,y,\xi):=y-f(x,\xi)= \left ( \textstyle\begin{array}{@{}c@{}} y_{1}-(x_{1}+\xi x_{1}x_{2})\\y_{2}-(x_{2}^{2}+\xi x_{1}) \end{array}\displaystyle \right ). $$

Let the parameter $\mu=0.01$. $(1,1)^{T}$, $(1,1)^{T}$ are initial points of Example 5.1. The numerical results are reported in Table 1, where ($x^{k}$, $y^{k}$) denotes the solutions of problem (2.3) and $(x^{*},y^{*})$ denotes the solution of problem (2.1). $D=\| (x^{k},y^{k})-(x^{*},y^{*})\|$.

**Table 1:** Computational results for Example 5.1

$N^{k}$	$x^{*}=(0.0105,0.0030)^{T}$, $y^{*}=(0.0089, 0.0094)^{T}$		
	$x^{k}$	$y^{k}$	*D*
10^3^	$(0.0115,0.0030)^{T}$	$(0.0091,0.0093)^{T}$	0.0010
10^4^	$(0.0114,0.0030)^{T}$	$(0.0090,0.0093)^{T}$	0.0009
10^5^	$(0.0112,0.0029)^{T}$	$(0.0089,0.0094)^{T}$	0.0007

### Example 5.2

Let *ξ* be the uniformly distributed random variable in $(0,1)$, and $F:R^{2}\times R^{2}\times\Omega\rightarrow R^{2}$ is defined as follows:

$$ F(x,y,z,\xi):= \left ( \textstyle\begin{array}{@{}c@{}} y_{1}-(x_{1}^{2}+\xi x_{1}x_{2}^{2})\\y_{2}-(\xi x_{2}^{2}+x_{1}) \end{array}\displaystyle \right ). $$

Let $(1,1)^{T}$, $(1,1)^{T}$ be the initial points of Example 5.2. The numerical results are reported in Table 2, where $(x^{k}, y^{k})$ denotes the solutions of problem (2.3) and $(x^{*},y^{*})$ denotes the solution of problem (2.1). $D=\| (x^{k},y^{k})-(x^{*},y^{*})\|$.

**Table 2:** Computational results for Example 5.2

$N^{k}$	$x^{*}=(1.8420, -0.4211)^{T}$, $y^{*}=(3.1497, 2.3370)^{T}$			
	$\mu^{k}$	$x^{k}$	$y^{k}$	*D*
10^3^	0.01	$(1.9321, -0.5234)^{T}$	$(3.2850, 2.5873)^{T}$	0.3155
10^4^	0.008	$(1.8564, -0.4328)^{T}$	$(3.1589, 2.4692)^{T}$	0.1338
10^5^	0.005	$(1.8462, -0.4281)^{T}$	$(3.1503, 2.3981)^{T}$	0.0616

## Results and discussion

In this paper, we give a reasonable reformulation for solving SSOCCP, which is called ERM model. To solve this ERM model, we pay attention to two problems: one is how to handle the property of non-differentiability, the other is how to obtain the value of expectation for the objective function. We employ a smoothing technique and a sample average approximation method to deal with the problems. Further, we show that the global solution and weak stationary point of this smoothing sample average approximation ERM problem converge to those of the true ERM model correspondingly.
